# The epidemiology and clinical features of personality disorders in later life; a study of secondary care data

**DOI:** 10.1002/gps.5837

**Published:** 2022-11-01

**Authors:** Natasha Treagust, Emad Sidhom, Jonathan Lewis, Chess Denman, Olivia Knutson, Benjamin R. Underwood

**Affiliations:** ^1^ University of Cambridge School of Clinical Medicine Cambridge Biomedical Campus Cambridge UK; ^2^ Department of Clinical Neurosciences University of Cambridge Cambridge Biomedical Campus Cambridge UK; ^3^ Windsor Unit Fulbourn Hospital Cambridgeshire and Peterborough NHS Foundation Trust Cambridge UK; ^4^ Department of Psychiatry University of Cambridge Forvie Site Cambridge UK

**Keywords:** later life, older adults, personality disorder

## Abstract

**Objectives:**

Personality disorders (PDs) are often conceptualised as impacting individuals throughout their life. However, there has been limited study of the disorders in those over the age of 65. We have used the psychiatric secondary care medical records of 21,971 individuals over the age of 65 from Cambridgeshire, UK, who received care between 2014 and 2021 to characterise older patients with a PD diagnosis.

**Methods:**

The data from all patients >65 with a diagnosis of personality disorder (PD) was extracted (*n* = 217) along with two comparison groups (*n* = 2170); patients <65 with a diagnosis of PD and patients >65 with a psychiatric diagnosis other than PD or dementia.

**Results:**

Compared to younger patients with PD, older patients were more likely to be male, married, suffering from a mixed PD and live in less deprived areas. Compared to patients >65 with diagnoses other than PD, older patients were more likely to be female, single or divorced and had a higher level of social deprivation. Our most striking finding was that older patients with PDs were more likely to experience polypharmacy. A mean of 18.48 different drugs had been prescribed over their lifetime, compared to 9.51 for patients >65 with other mental health diagnoses.

**Conclusion:**

Here we present the largest ever description of this group of patients and provide insights that could inform clinical practice and future research.

## Key points

- Using the electronic healthcare records of a population of nearly 1 million people, we have shown patients >65 with a diagnosis of personality disorder (PD) to be a group with distinct characteristics compared to younger patients with PD and older patients with other mental health diagnoses.
- The number receiving care is much smaller than expected based on studies of the general population, which may indicate unmet need.
- Patients in this group show improvement in their Health of the Nation Outcome Scales scores following treatment in NHS mental health services.
- Older people with a diagnosis of PD received double the number of diagnoses and different prescription medications in their lifetime compared to patients of the same age with other psychiatric diagnoses, raising the possibility that interventions such as a medication review might be helpful.

## 1 INTRODUCTION

Personality disorder (PD) encompasses a group of diagnoses where an individual's thoughts, feelings and emotions deviate significantly from the expectations of society.^1^ These characteristics must be present over a prolonged period, independent of other physical or mental health diagnoses. These disorders impair an individual's ability to function, for many leading to significant distress and a concomitant increased likelihood of experiencing further mental disorder and premature mortality.^2^ Individuals diagnosed with PD often have above‐average levels of interaction with healthcare services which potentially increases their risk of iatrogenic harm and has a substantial economic cost.^3^ There is an overlap between the concept of PD and other clinical constructs, including complex emotional needs. In this paper we use the term PD as the data here are based on that diagnostic classification. Similarly, we recognise that diagnostic categorisation of personality has been questioned, for example, in contrast to a dimensional/trait approach but in this paper we have used ICD diagnostic categories to identify patients as that is how the clinical records are categorised.

The prevalence of PDs in the general population of western countries was reported as 12.16% by Volkert and colleagues in a systematic review and meta‐analysis.^4^ However, the average age of patients in the 10 papers reviewed was 33–51 years old. Few patients over the age of 65 were included meaning this group are relatively poorly described. Whilst some have looked at specific associations, for example, between older people with PD and quality of life,^5^ not all sub‐types of PD were included, and the majority of patients were not over 65. More recent systematic reviews, for example, by Penders and colleagues,^6^ have highlighted the urgent need for more research concerning the epidemiology of this patient group.

Given the paucity of current research into PDs in later life, we sought to characterise this patient group and compare them to younger patients with PDs and older patients with other psychiatric diagnoses using a large clinical database from an NHS clinical service.

## 2 MATERIALS AND METHODS

We conducted an observational study of patients with a diagnosis of PD under the care of Cambridge and Peterborough NHS Foundation Trust (CPFT) mental health services. The study uses anonymised data from the electronic records of patients who received care from CPFT between 2014 and 2021. CPFT serves a population of approximately 1 million people of which 165,000 are over 65. The CPFT research database (NHS research ethics reference 17/EE/0442) uses CRATE (clinical records anonymisation and text extraction) software to anonymise records and associate them with a random patient‐specific identifier and contains the records of 21,971 individuals over the age of 65. The database has overarching ethical approval and this specific project was further approved by the database oversight committee. The initial information was collected by the electronic patient record used by the trust during the period (Servelec RiO electronic care record system).

To identify patient over the age of 65 with a diagnosis of PD, we searched the database using Structured Query Language queries with the criteria of being treated between 2014 and 2021, being older than 65 and having an ICD‐10 diagnosis of F60‐62, which generated a patient population of *n* = 217.

The patients identified were then compared to two 10 times larger control groups whose data was extracted using the same methodology. These groups were patients under 65 treated by CPFT with a PD diagnosis (*n* = 2170) and patients over 65 treated by CPFT with a psychiatric diagnosis other than PD or dementia (*n* = 2170). The groups were both unmatched samples from the overall patient population. Patients with dementia were excluded from all groups. Demographic features of patients over 65 with PDs were also compared to those over 65 in the general population using publicly available 2011 census data for Cambridgeshire and Peterborough.^7^

The index of multiple deprivation (IMD) was used as an indicator of socioeconomic status. This is a national system compiled by dividing England into lower‐layer super output areas each with a population of approximately 1500. The areas are ranked based on scores in seven domains from 1, the most deprived, to 32,844, the least.

We used the Health of the Nation Outcome Scales (HoNOS) to indicate the psychiatric health of our patients before and after treatment. This enabled analysis of treatment outcomes using paired initial and discharge assessments. Not all the patients had been discharged during the period of the study and, of those who had been discharged only some had received an initial HoNoS assessment. To enable paired analysis, manual extraction of pairs was completed. A pair was defined as the earliest initial assessment coded ‘initial’ combined with the earliest HoNoS assessment coded ‘discharge’ to follow this. The pairs were then analysed using a paired two‐sample *t*‐test assuming equal variance.

The descriptive statistics calculated were mean, median, mode and standard deviation. Chi‐squared tests were conducted to assess the significance of the differences between groups in categorical data, such as marital status, PD diagnosis and IMD decile. To test the significance of the difference between average HoNOS scores, unpaired *t*‐tests were used for between‐group comparisons and paired *t*‐tests for within groups comparisons.

## 3 RESULTS

The database contained *n* = 21,971 records of patients over 65, and *n* = 217 met our inclusion criteria. This represents 0.98% of the patients seen over the 2014–2021 period and 0.138% of the projected population of Cambridgeshire and Peterborough over 65 in 2020.

**FIGURE 1:**
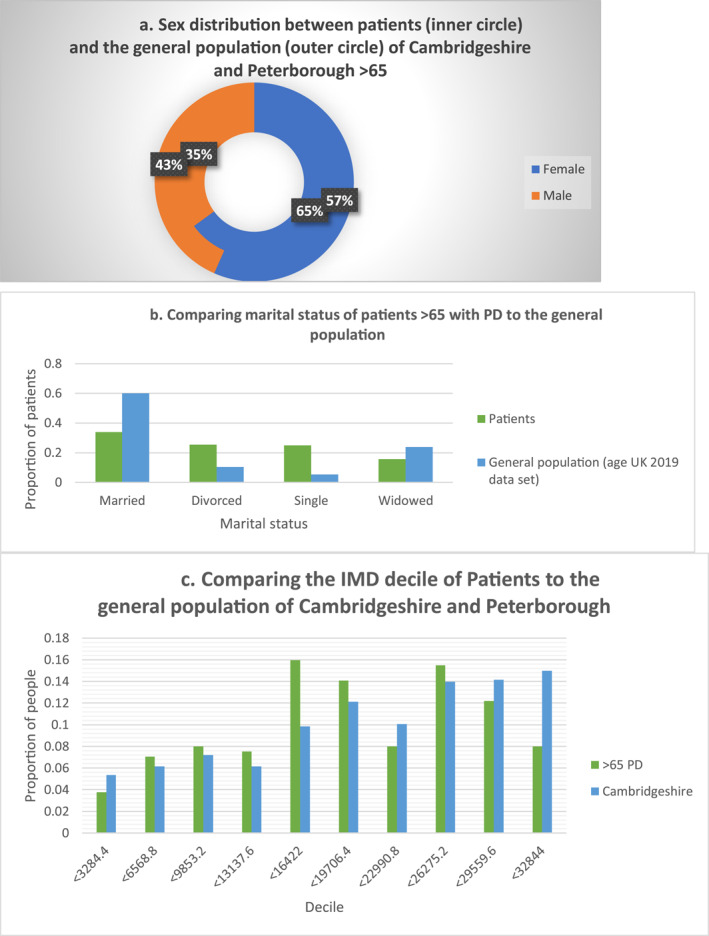
The characteristics of patients >65 with a diagnosis of PD compared to the general population of Cambridgeshire. (A) Comparing the sex distribution between patients >65 and the general population of Cambridgeshire >65. (B) Comparing the marital status of patients >65 and the general population of Cambridgeshire >65. (C) Comparing the deprivation index of patients >65 and the general population of Cambridgeshire. PD, personality disorder

The comparison of patients over 65 with a diagnosis of PD to the general population of Cambridgeshire and Peterborough is represented in Figure 1. Our patients had a mean age of 75.25 (SD = 7.72). 65.0% were female (*n* = 141) average age 75.38 (SD = 7.86) and 35.0% male (*n* = 76) with an average age of 75.03 (SD = 7.52). When compared to the Cambridgeshire and Peterborough data for the over 65 s of which 46.1% are male, this distribution was found to be significantly different (*χ* ^2^ = 30.1, d.f = 1, *p* < 0.001). 33% of the patients were married, 25% divorced and 15% widowed. As illustrated in Figure 1B, this is significantly different to the proportion in the general population >65 reported in the 2017 office for national Statistics estimates of marital status and living arrangements data set^8^ (*χ* ^2^ = 33.92, d.f. = 3, *p* < 0.001). In the general population, a much greater percentage are married.

Analysis of the distribution of IMD also indicates patients over 65 with a diagnosis of PD are more deprived than the general population in absolute terms. However this difference in distribution did not reach statistical significance (*χ* ^2^ = 13.65, d.f = 9, *p* > 0.1).

**FIGURE 2:**
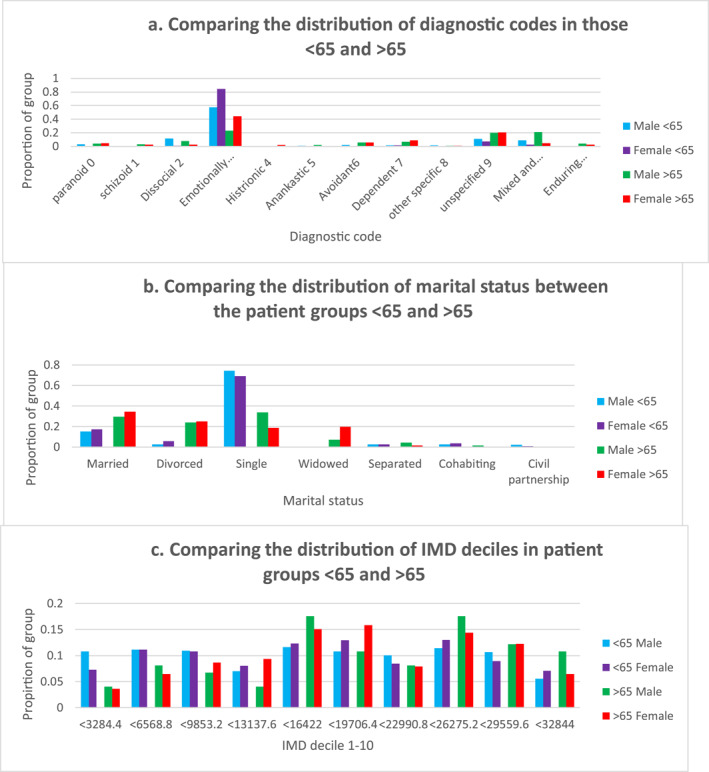
Comparison of diagnostic subgroups. (A) Comparing the distribution of diagnoses in patients with PD >65 and <65. (B) Comparing the distribution or marital status within patient groups <65 and >65. (C) Comparing the distribution of IMD deciles within in patient groups <65 and >65. IMD, index of multiple deprivation; PD, personality disorder

Patients received a broad range of diagnoses with at least one from each of the ICD10 PD subtypes. The most frequently coded was F60.3, emotionally unstable personality disorder (EUPD) (*n* = 90). When stratifying by gender, F60.3 remained the most frequently coded. Nevertheless, there was a significant difference in the distribution of diagnoses between gender (*χ* ^2^ = 29.24, d.f = 11, *p* < 0.001). Amongst women (*n* = 156), 44% of PD diagnoses coded were F60.3 but only 23% (*n* = 90) in men. This is illustrated in Figure 2A. Mixed and other PDs comprised 21% of coded diagnoses in men compared to 4% in women.

Our patient group had received an average of 3.95 (SD = 3.16) coded diagnoses in their lifetimes and 18.49 (SD = 13.25) different prescription medications.

**FIGURE 3:**
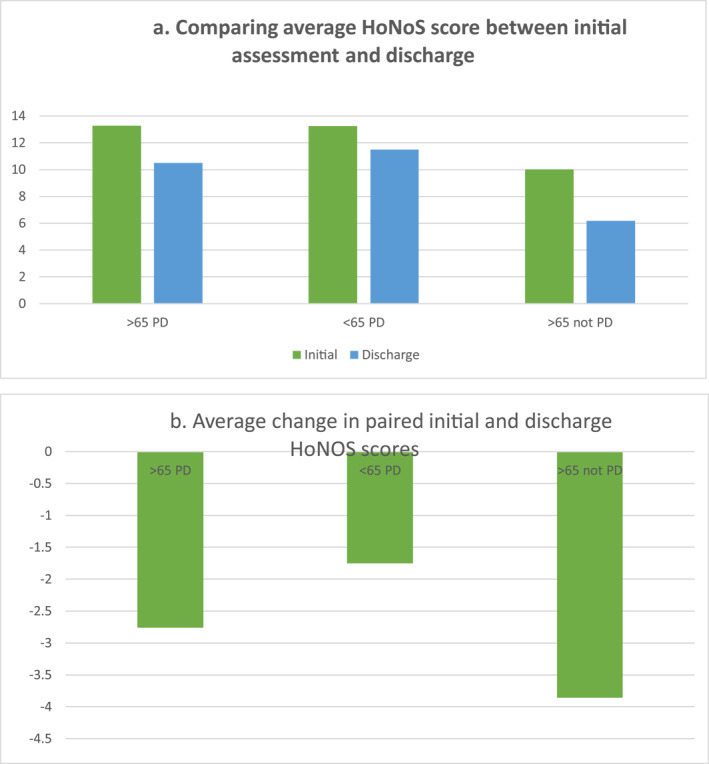
HoNOS Scores. (A) Comparing the average HoNoS scores of patients on initial assessment and discharge between the three groups studied. (B) Comparing the average change in patients HoNoS scores from initial CPFT assessment to discharge between the three groups studied. CPFT, Cambridge and Peterborough NHS Foundation Trust; HoNOS, Health of the Nation Outcome Scale

The outcome of secondary care treatment was evaluated using HoNoS scores. Eighty five paired records were identified where scores were taken on initial assessment and at discharge. There was a significant difference between these on a paired two‐sample test for total HoNoS scores *p* = 0.0004 with lower scores on discharge suggesting a positive effect of intervention by the mental health services. This is illustrated in Figure 3A,B.

### 3.1 Comparison 1: Patients under 65 with a diagnosis of F60‐62

A 10 times larger sample of *n* = 2170 of patients under 65 was extracted for comparison. This was 69.5% of the under 65 s in the database with a PD diagnosis (*n* = 3121). The group had a mean age of 37.4 years old (SD = 11.39).

There was no significant difference between the gender distribution of the patient groups (*χ* ^2^ = 2.17, d.f = 2, *p* > 0.05). In both samples, most patients with a PD diagnosis were female, 69.1% of those under 65% and 65.0% of those over 65.

There was a significant difference in marital status (*χ* ^2^ = 417.81, d.f = 6, *p* < 0.001). As shown in Figure 2B a greater proportion of patients under 65 were single, 70.7% of the total compared to 24.2% of those over 65, Figure 2B. The analysis of deprivation indicates younger patients live in more deprived areas. 30.3% of the under 65 s have postcodes from the bottom three deciles compared to 18.7% of those over 65, this is shown in Figure 2C. However, this difference was not found to be significant in a chi‐squared test (*χ* ^2^ = 16.02, d.f = 9, *p* > 0.05). We also found marked differences in the distribution of PD subtypes. Proportionally more patients under 65 had a diagnosis of F60.3 (75.8% vs. 37%) and other PD diagnoses were less common compared to our older patient group (Figure 2A).

The average number of different prescription medications over the lifetime for patients under 65 was 12.57 (SD = 11.47). This is significantly less than the average 18.48 given to the over 65 s (*t* = 7.139, *p* < 0.001). However, they also have fewer lifetime diagnoses, an average of 2.78 (SD = 2.60) (*t* = 6.16, *p* < 0.001).

The average HoNoS scores given to under 65 s on initial assessment were no different from the initial scores taken for those over 65 suggesting they present to secondary care with equal levels of need (*t* = 0.9663, *p* > 0.05). In the patients where paired initial and discharge HoNoS were calculated, both the under 65 s and over 65 s had a significant reduction in their score after treatment (paired *t*‐test. <65 *t* = 4.1984, >65 *t* = 6.275). There was an average 1.01 points greater improvement in HoNoS score after‐treatment in the over 65 s (2.26 vs. 1.75). However, the magnitude of improvement in the two groups was not significantly different (unpaired *t*‐test [*t* = 1.31, *p* > 0.05]).

### 3.2 Comparison 2: Patients >65 with a diagnosis other than F60‐62 and F0

**FIGURE 4:**
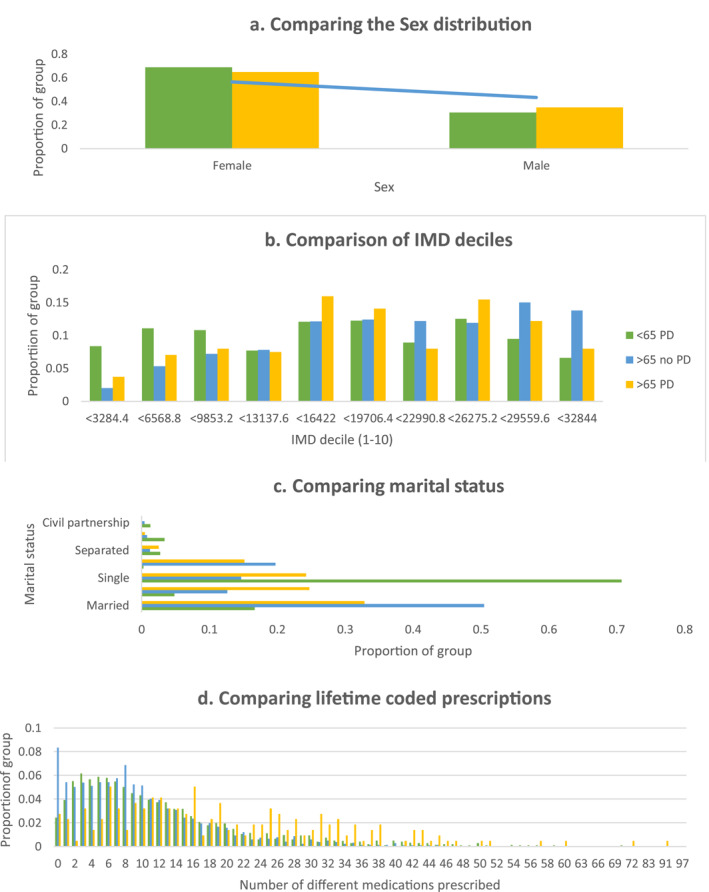
Comparison of those with a PD diagnosis versus those <65 with a diagnosis of PD and those >65 with a diagnosis other than PD or Dementia. (A) Comparing the sex distribution within the groups. (B) Comparing the marital status of the groups. (C) Comparing the deprivation index of the groups. (D) Comparing lifetime prescriptions coded in Rio between the groups. PD, personality disorder

An additional 10 times larger sample of patients over 65 with psychiatric diagnoses other than PD or dementia *n* = 2170 was extracted for comparison. The gender distribution and marital status of this group differed significantly from patients with a PD diagnosis. The patients with an alternative diagnosis contained a greater proportion of men (43% vs. 35%). As shown in Figure 4C, a much greater proportion was married, 50% compared to 33% of individuals with a PD diagnosis (*χ* ^2^ = 73.23, d.f = 6, *p* < 0.001).

IMD analysis indicated patients with a PD diagnosis lived in more deprived areas than those with other psychiatric diagnoses (*χ* ^2^ = 17.38, d.f = 9, *p* < 0.05). In terms of the health of these individuals, there were significant differences both in the lifetime diagnoses coded on the system and the number of different prescription medications they had received. Those with alternative diagnoses had on average received 9.51 (SD = 8.33) different prescription medications, almost half that of those with a PD diagnosis, and 1.52 (SD = 1.13) diagnoses, again around half the average number coded for individuals with a PD diagnosis.

## 4 DISCUSSION

Our study is one of the first to analyse the secondary care data of those over 65 with a diagnosis of PD and to our knowledge is the largest in terms of the number of patients included. Several findings may resonate with clinician experience but previously have had a limited evidence base.

One of the most striking findings was the small number of patients aged over 65 with a diagnosis of PD. The group is much smaller than expected based on the prevalence in the general population over 65 reported in other studies. For example, Reynolds and colleagues^9^ used data from a large survey of a nationally representative sample of Americans and found the prevalence of PDs to be 12.6% in the over 65 s. Applying this to the projected Cambridgeshire and Peterborough population over 65 in 2020 (*n* = 157,815), we would predict almost 20,000 residents of Cambridgeshire over the age of 65 to be living with a PD. Our patient group is 1% of this figure.

There are several possible explanations for this. As people age, they may develop coping strategies or build social scaffolding which mitigates the disorder's impact on their daily life and consequently may be less likely to present to secondary care. However others have argued that PD symptomatology changes in later life.^10^ This does not necessarily mean becoming less severe but could impact the frequency with which patients are seen by healthcare professionals or diagnosed. This might be consistent with a longitudinal study of individuals with borderline PD which found older adults had more severe depressive and anxiety symptoms while younger adults had a higher incidence of suicide attempts and aggression.^11^ Alternatively, the low numbers could reflect some of the impediments to diagnosing older people with a PD. One contributor to this is the increased likelihood of our older patients having comorbid physical and mental health conditions.^12^ Disentangling the signs and symptoms of these conditions increases the challenge of making a definitive diagnosis of a PD. Finally, there may be reluctance in diagnosing PD on the part of clinicians working in old age psychiatry. From a clinical perspective a comprehensive psychological formulation is likely to inform and enhance care planning and may be easier for patients to accept than strict diagnostic categories, but may also contribute to lower rates of diagnostic coding.

The disparity in numbers between the under and over 65 s with a PD diagnosis is also notable. A PD is a diagnosis associated with increased mortality, and it may be some patients do not live long enough to move into old age services.^13^ Alternatively, a PD may improve over time meaning individuals do not need secondary care. However, the disparity could also be explained by there being a large unmet need of patients not being seen by services or individuals who are assessed being given diagnostic codes for other comorbid disorders (for example affective disorder) rather than a PD. These possibilities require further examination.

Patients over 65 with PDs differed in almost all aspects when compared to younger patients or older patients with other psychiatric diagnoses. Marital status differed significantly when the patient group was compared to both comparator groups. As would be expected when comparing young and old with PDs, there is a transition from a high proportion of single people to a larger proportion who are married, divorced or widowed. The greater number of years lived gives more time for these events to occur. However, in the over 65 s with PD, the married group is smaller, and the divorced or single groups are larger compared to both patients over 65 without PD and the general population over 65. This finding may reflect difficulty in sustaining relationships.

We also found differences in types of PD diagnosed in older compared to younger patients and a different profile of deprivation index. Older patients were less likely to be diagnosed with EUPD, though this remained the single largest diagnostic category, with relatively more mixed and unspecified PD in older patients. The fact that EUPD is still the most common diagnosis may be because some features of EUPD result in more frequent contact with healthcare providers (for example following self‐harm), differences in perceived face validity of diagnostic categories or some patients being diagnosed and coded with other disorders (for example obsessive compulsive disorder in anankastic PD) resulting in lower rates of other PD diagnosis. Older patients with PDs had an IMD distribution indicative of lower levels of deprivation compared to younger patients, but higher levels compared to older people with affective disorders. Reassuringly we found significant improvement in HoNOS scores in older patients with PDs being treated in the service which is counter to the therapeutic nihilism sometimes encountered in both the treatment of PDs and in older adults.

The most striking difference found between our groups was the lifetime number of different medications prescribed. As expected, those over 65 have received significantly more than those under 65. However, when compared to those over 65 with a diagnosis of affective disorder, those with a PD diagnosis have received almost double the number prescribed to their age‐matched counterparts and had significantly more diagnoses recorded. The greater number of medications may be explained by increased morbidity. Alternatively, it may be that some of these prescriptions are not clinically appropriate, or no longer appropriate if they have not been reviewed for some time, particularly as drug treatment for PD per se is unsupported by the current evidence base or NICE treatment guidelines and highlights the importance of continued medication review, especially at transitions of care.^14^ This area should be further explored as medication side effects exacerbate with age and a review of medication in this patient group may provide a simple intervention to minimise harm. The importance of resolving this is highlighted by papers such as that of Huang and colleagues^15^ who studied the mortality risk of specific groups of medications in older adults with polypharmacy. They found the mental health drug cluster to be the only pattern investigated associated with an increase in all‐cause and cardiovascular disease mortality. This raises the possibility that a simple intervention of medication review could benefit this patient group.

While the analysis that forms the basis of this paper endeavoured to produce the most comprehensive characterisation of this patient group to date, we acknowledge there are limitations. The first of these arises from only having 217 patients over 65 with a PD diagnosis within our records. To mitigate this, we included patients from the full timespan covered by our database. To advance our work future studies could pool data from mental health trusts to assemble a greater sample size.

The second limitation of our work occurs due to the nature of the database we have used. Text‐based extraction allows us to make inferences and form hypotheses based on comparisons to younger patients and those with conditions other than PDs. However, to take this work further interventional studies are needed to test the hypothesises and assess if the differences we have found can be used for the benefit of patients. A good example of this is the significantly higher number of different medications prescribed to individuals with PDs over their lifetime. From this, we infer they may be at a higher risk of iatrogenic harm. This hypothesis can only be fully explored through additional research such as qualitative analyses of individual experiences (including the meaning and value patients attribute to their medication), examination of where patients are cared for (primary or secondary care and what each setting might require to deliver care differently) or direct interventions to assess the effect of reducing medication burden.

In conclusion, we have shown individuals over 65 with a PD diagnosis to be a patient group distinct from younger patients with PD or older patients with other psychiatric diagnoses. The current lack of research on these patients is an impediment to providing high‐quality evidence‐based care, though our data suggests that current interventions are associated with improvement as measured by the HoNOS. The small size of our group indicates there may be a significant number of individuals in the community who are undiagnosed. The potential consequences of this include misdiagnosis, a hindrance to treatments for comorbidities and missing out on the chance to receive effective treatment for their disorder. The high levels of polypharmacy identified is especially concerning and further research should concentrate on establishing to what extent this prescribing is appropriate and whether simple interventions can rationalise prescribing to maximise benefit and minimise harm.

## AUTHOR CONTRIBUTIONS

**Benjamin R. Underwood**: Conceptualisation; Methodology; Resources; Supervision; Writing: Review and Editing; Project administration. **Natasha Treagust**: Methodology; Formal analysis; Investigation; Writing – Original draft. **Chess Denman**: Conceptualisation; Writing: Review and Editing. **Emad Sidhom**: Investigation; Visualization; Writing: Review and Editing. **Jonathan Lewis**: Software; Data Curation; Writing: Review and editing. **Olivia Knutson:** Conceptualisation.

## CONFLICT OF INTEREST

The author declares that there is no conflict of interest that could be perceived as prejudicing the impartiality of the research reported.

## Data Availability

The data that support the findings of this study are available on request from the corresponding author. The data are not publicly available due to privacy or ethical restrictions.
